# Gross tumor volume delineation in primary prostate cancer on ^18^F-PSMA-1007 PET/MRI and ^68^Ga-PSMA-11 PET/MRI

**DOI:** 10.1186/s40644-022-00475-1

**Published:** 2022-07-22

**Authors:** Yan-Nan Zhang, Zhen-Guo Lu, Shuai-Dong Wang, Xin Lu, Lei-Lei Zhu, Xu Yang, Li-Ping Fu, Jun Zhao, Hai-Feng Wang, Zuo-Lin Xiang

**Affiliations:** 1 Department of Radiation Oncology, Shanghai East Hospital, Tongji University School of Medicine, 200120 Shanghai, China; 2 Department of Urology, Shanghai East Hospital, Tongji University School of Medicine, Shanghai, 200120 China; 3 Department of Nuclear Medicine, Shanghai East Hospital, Tongji University School of Medicine, Shanghai, 200120 China

**Keywords:** Prostate cancer, Gross tumor volume, Radiotherapy, PSMA PET/MRI

## Abstract

**Background:**

We aimed to assess the clinical value of ^18^F-PSMA-1007 and ^68^Ga-PSMA-11 PET/MRI in the gross tumor volume (GTV) delineation of radiotherapy for prostate cancer (PCa).

**Methods:**

Sixty-nine patients were retrospectively enrolled (57 in the ^18^F subgroup and 12 in the ^68^Ga subgroup). Three physicians delineated the GTV and tumor length by the visual method and threshold method with thresholds of 30%, 40%, 50%, and 60% SUVmax. The volume correlation and differences in GTVs were assessed. The dice similarity coefficient (DSC) was applied to estimate the spatial overlap between GTVs. For 51 patients undergoing radical prostatectomy, the tumor length (Lpath) of the maximum area was measured, and compared with the longest tumor length obtained based on the images (L_MRI_, L_PET/MRI_, L_PET_, L_PET30%_, L_PET40%_, L_PET50%_, L_PET60%_) to determine the best delineation method.

**Results:**

In the ^18^F subgroup, (1) GTV-PET/MRI (*p* < 0.001) was significantly different from the reference GTV-MRI. DSC between them was > 0.7. (2) GTV-MRI (*R*^*2*^ = 0.462, *p* < 0.05) was the influencing factor of DSC. In the ^68^Ga subgroup, (1) GTV-PET/MRI (*p* < 0.05) was significantly different from the reference GTV-MRI. DSC between them was > 0.7. (2) There was a significant correlation between GTV-MRI (*r* = 0.580, *p* < 0.05) and DSC. The longest tumor length measured by PET/MRI was in good agreement with that measured by histopathological analysis in both subgroups.

**Conclusion:**

It is feasible to visually delineate GTV on PSMA PET/MRI in PCa radiotherapy, and we emphasize the utility of PET/MRI fusion images in GTV delineation. In addition, the overlap degree was the highest between GTV-MRI and GTV-PET/MRI, and it increased with increasing volume.

## Introduction

Prostate cancer (PCa) is the most common cancer among men worldwide and the second most common cause of cancer-associated death after lung cancer. The incidence of PCa is increasing annually, ranking first in cancer as harmful to men's health due to early diagnosis and advances in treatments [1]. Except for radical prostatectomy and brachytherapy, external beam radiation therapy (EBRT) alone or in conjunction with androgen deprivation therapy (ADT) is a significant curative therapy for PCa patients [2]. In recent years, conventional radiotherapy has been gradually replaced by precise radiotherapy, which requires accurate clinical staging and target delineation. Biochemical disease control may improve through dose escalation to the pivotal lesions, but increasing the dose to the entire prostate is not preferable and the maximal dose is limited due to toxicity [3]. Hence, it is critical to precisely delineate the target volume to increase the therapeutic focus on dominant intraprostatic lesions and reduce toxicity to adjacent tissues.

Currently, biparametric magnetic resonance imaging (bpMRI) examination is the gold standard imaging modality for the diagnosis, staging, and gross tumor volume (GTV) delineation of PCa [4]. Functional imaging, which can elucidate the metabolic characteristics of tumors, has been increasingly applied in radiotherapy applications. In the context of this development, positron emission tomography (PET) imaging, as a prosperous imaging modality, plays a significant role in the treatment of PCa [5]. A hybrid PET/MRI system integrates the metabolic imaging capabilities of PET, providing accurate morphological assessment and higher spatial resolution of MRI [6], thus allowing more complete information to be obtained for clinical use and more accurate PCa tumor localization than MRI or PET alone [7]. There have been some reports of GTV delineation of malignant tumors based on PET/MRI in radiotherapy. GTV delineation in radiotherapy with state-of-the-art MRI combined with PET, especially in patients with head and neck cancer, cervical cancer, and prostate cancer, may be of beneficial.

Prostate-specific membrane antigen (PSMA) is a type II transmembrane glycoprotein that is selectively overexpressed in almost all PCa cells, especially in aggressive, poorly differentiated, and metastatic PCa [8]. Over the past decade, the rapid development of PSMA PET has enhanced its clinical application in the diagnosis, staging, and detection of therapeutic efficacy for PCa. Recently, ^68^Ga-PSMA-11 has also demonstrated a promising detection capability for high-risk diseases and has been increasingly employed in routine clinical practice [9]. However, a large proportion of patients with locally recurrent disease cannot be distinguished from other patients by urinary tracer excretion. ^18^F-PSMA-1007, which has very low urine clearance, has shown promising usefulness in clinical treatment management and may aid clinicians in making appropriate decisions [10].

To our knowledge, GTV delineation of PCa based on PSMA PET/MRI in radiotherapy planning has seldom been reported, and most of these studies focus on PSMA PET/CT. Consequently, we propose the assumption that GTV delineation based on PSMA PET/MRI is feasible and could improve delineation accuracy simultaneously. The objective of this study was to assess the value of the clinical application of PSMA PET/MRI in the GTV delineation of radiotherapy for PCa.

## Materials and methods

### Patients

The inclusion criteria were as follows: age over 18 years; no other metastases; no treatment priori to undergoing the PET/MRI scans; and good cooperation during the scanning procedure, resulting in high-quality diagnostic images and tumor volume delineation. Ultimately, 69 patients with PCa were retrospectively recruited between May 2020 and April 2021. All participants provided written informed consent. This study involving human participants was carried out in accordance with the principles of the 1964 Declaration of Helsinki.

### PET/MRI image acquisition

All patients needed adequate hydration and received an intravenous injection of ^18^F-PSMA-1007 or ^68^Ga-PSMA-11 (2.96–3.7 MBq/kg) 1 h before undergoing a PET scan on the PET/MRI system (United Imaging Health care, Shanghai, China). Because the high radioactivity of the urinary system may have led to halo artefacts and false-positive results, each patient was instructed to empty his bladder to reducing potential artefacts.

During reconstruction, the system's PET component uses Time of Flight (TOF) technology to display images, reducing noise and improving sensitivity, resulting in improved image quality. The PET image was reconstructed by the ordered subsets expectation maximization (OSEM) algorithm (including 2 iterations, 20 subsets, a 4 mm full width at half maximum (FWHM) Gaussian filter, and a 150 × 150 image matrix).

The hybrid system has a built-in MRI system that can generate a magnetic field intensity of 3 T. MRI scans, including T1WI imaging, T2WI imaging, and DWI (b = 100, 500, 1000, 1500 s/mm^2^) of the prostate and pelvis were adopted simultaneously. The apparent diffusion coefficient (ADC) was calculated by DWI. T1WI sequence parameters were as follows: repetition time (TR)/echo time (TE) = 5.04/2.24 ms, 4 mm slice thickness, 20% interslice gap, 350 mm × 350 mm field of view (FOV), and a 256 × 256 matrix. The axial FSE T2WI sequence parameters were as follows: TR/TE = 3998/88.74 ms, 6 mm slice thickness, 20% interslice gap, 300 mm × 300 mm FOV, and a 320 × 320 matrix.

### GTV delineation

When interpreting PET images, uncorrected and attenuation-corrected images were also evaluated simultaneously to confirm the presence of artefacts (e.g., contrast, metal graft, or patient changes in position). At the same time, it was necessary to adjust the display window width and window level of the image to accommodate the PSMA ligand uptake site or its adjacent high-uptake organs (e.g., kidney, ureter, or bladder). Zamboglou et al. [11] showed that using the same window level (SUVmin-max: 0–5) can reduce variation between observers in GTV delineation based on ^68^Ga-PSMA PET and inferred that setting a fixed optimal window level may also lead to increased consistency in ^18^F-PSMA PET. Therefore, a uniform fixed window position was used for each observer in this study to avoid bias between observers. The interpretations of all images were confirmed by an experienced physician specializing in nuclear medicine. Three board-certified radiation oncologists (A, B, C) with experience in PCa verified the images and independently delineated the target of all suspected visible tumors. Lymph nodes were not delineated in our study. In this case, GTVs based on MRI, PET, and PET/MRI were available for each patient.

During the GTV-MRI delineation process, all physicians combined T1WI, T2WI, and DWI images (including ADC images) to determine the lesions sites. PCa lesions were relatively hypointense on T1WI images, were relatively equal in hypointense on T2WI images, and demonstrated high signal intensity on DWI images with low ADC values. After the exclusion of inflammatory lesions or other benign lesions, PCa was determined by integrated multisequence images. Eventually, the GTV-MRI was obtained by manual contouring on axial T2WI MRI by radiation oncologists. L_MRI_ was obtained by measuring the maximum diameter of the maximum axis plane of the target area.

PSMA PET images were contoured in two ways: (1) GTV-PET_VIS_ was delineated on PET images by the visual method. When PSMA uptake was significantly higher than that in the surrounding normal tissue background, we considered this to be an indicator of metabolic abnormalities and suspected lesions. L_PET_ was obtained by measuring the maximum diameter of the maximum axis plane of the target area. (2) GTV-PET_30%/40%/50%/60%_ values were respectively obtained by the contour method with SUVmax thresholds of 30%, 40%, 50%, and 60%. PSMA uptake was quantified using the standardized uptake value (SUV). Next, the percentage of SUV in each voxel was determined by measuring the highest SUV in each corresponding prostate. L_PET30%_, L_PET40%_, L_PET50%_, and L_PET60%_ were obtained by measuring the maximum diameter of the maximum axis plane of the target area.

**Fig. 1:**
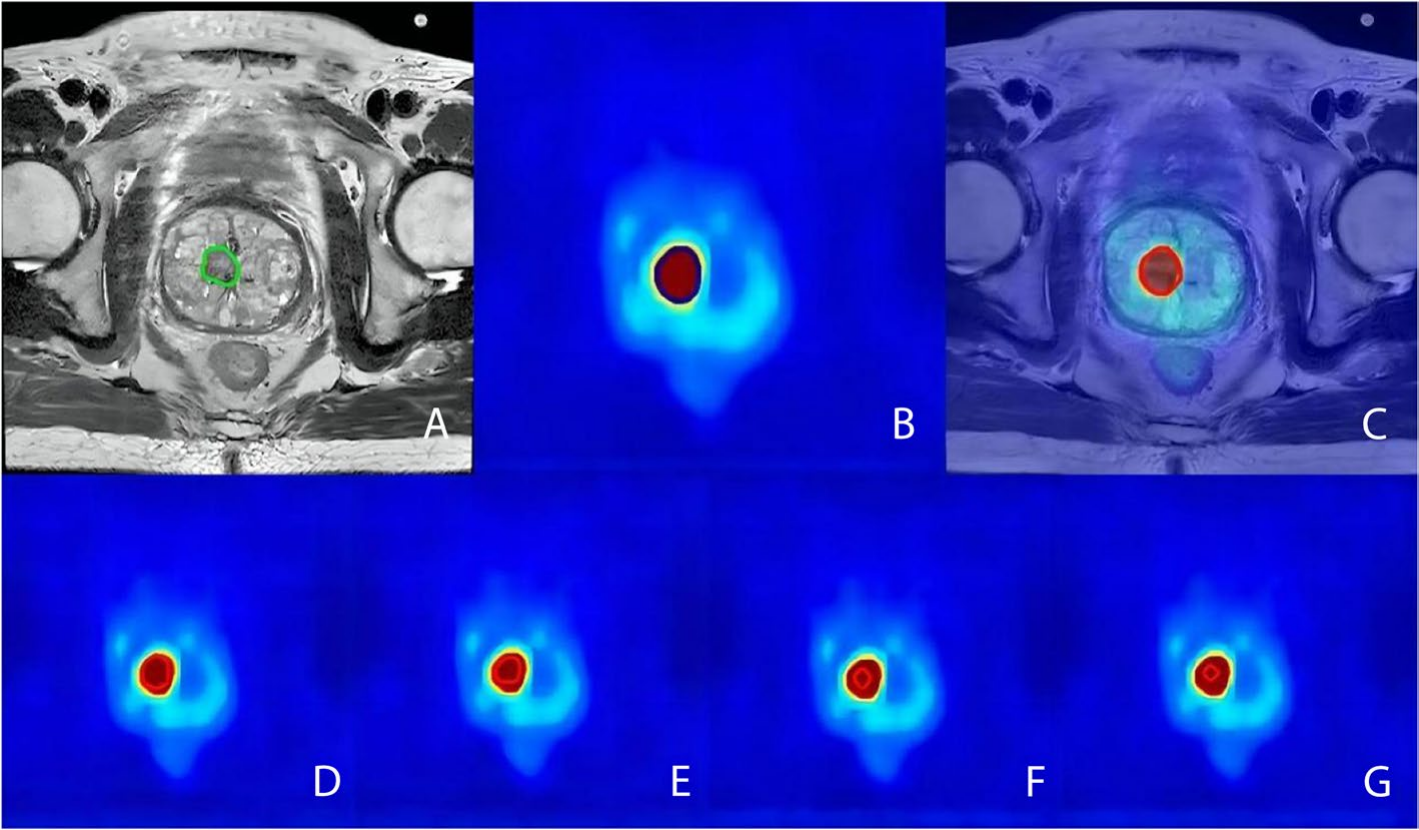
A 70-year-old male with prostate cancer in the ^18^F subgroup. The green line represents GTV-MRI (**A**). The blue line represents GTV-PET (**B**). The red line represents GTV-PET/MRI (C). The red line represents GTV-PET_30%_, GTV-PET_40%_, GTV-PET_50%_ and GTV-PET_60%_ (**D**, **E**, **F**, and **G**, respectively)

**Fig. 2:**
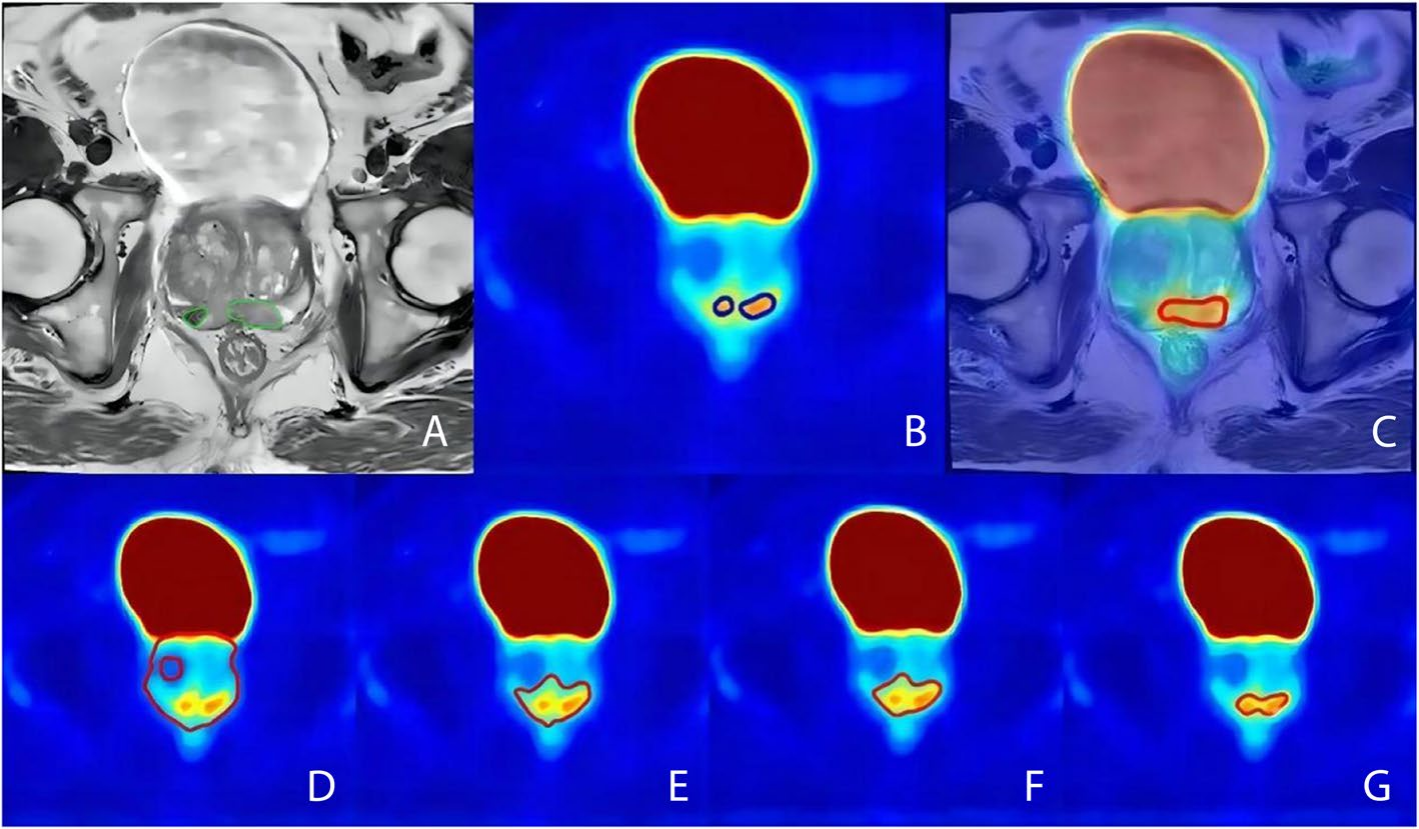
A 78-year-old male with prostate cancer in the ^68^Ga subgroup. The green line represents GTV-MRI (**A**). The blue line represents GTV-PET (**B**). The red line represents GTV-PET/MRI (**C**). The red line represents GTV-PET_30%_, GTV-PET_40%_, GTV-PET_50%_, and GTV-PET_60%_ (**D**, **E**, **F**, and **G**, respectively)

The GTV-PET/MRI was obtained by manual contouring following the fusion of the PET images with the T2WI axial images. The abnormal lesions with higher local radioactive uptake in the prostate gland than that in adjacent normal tissues and the above-described MRI characteristics were judged as positive lesions. L_PET/MRI_ was obtained by measuring the maximum diameter of the maximum axis plane of the target area. PSMA PET-based vs. conventional imaging-based examples are shown in Figs. 1 and 2.

### Pathological specimen measurement

A total of 51 patients received radical prostatectomy within one month after PET/MRI scanning, including 40 patients in the ^18^F subgroup and 11 patients in the ^68^Ga subgroup. The average interval between PET/MRI examination and surgery for these patients was 20 days. Due to the slow growth rate of PCa tumor cells, tumor growth during this period could be ignored. In other words, the size of the specimen could represent the size of the tumor on PET/MRI examination. The isolated surgical specimens were completely immersed in formalin for more than 24 h. After fixation, the largest layer of tumor tissue was sliced. Then, the tissue section was fixed on a small board, and the longest tumor length at this layer (Lpath) was measured with a Vernier caliper. Lpath is the gold standard to used to compare the tumor length measured by different methods. This comparative modality has been adopted in previous studies [12, 13].

### Analysis

The median (IQR) was used for obtained GTVs. The dice similarity coefficient (DSC) values were used to evaluate spatial overlap within GTV-MRI, GTV-PETs, and GTV-PET/MRI. The calculation formula for DSC was 2 × (A ∩ B)/(A + B), where A and B represent two volumes, (A ∩ B) represents the volume of the intersection, and (A + B) represents the absolute sum of their volumes [14]. The range of the DSC is 0–1. The DSC value is 1 when the two tumor lesions overlap completely in space and 0 when there is no overlap between them. The higher the DSC value is, the higher the overlap degree. It is generally recognized that the coincidence degree is better when the DSC is greater than 0.7 [15].

Statistical analyses were performed using SPSS, Version 24.0 (IBM Corp., Armonk, NY, USA) and GraphPad PRISM, Version 8.0 (GraphPad Software, San Diego, CA). One-way analysis of variance (ANOVA) was performed to assess the differences among the observers. A nonparametric paired-sample Wilcoxon signed-rank test was carried out to compare GTV-MRI with GTV-PET and GTV-PET/MRI. Spearman analysis was used to perform the correlation between GTV-MRI and GTV-PETs, GTV-MRI and GTV-PET/MRI, DSC and clinical parameters (including age, the reference GTV-MRI, PSA, Gleason score, risk classification, and SUVmax). Multiple linear regression was performed to determine the influencing factors of DSC at a statistical significance threshold of < 0.05. Spearman analysis was used to determine the correlation between Lpath and the tumor length measured by different methods. The intra-class correlation coefficient (ICC) and Bland Altman plot were used to evaluate the consistency of the different methods with the gold standard pathological methods. The value of ICC is between 0 and 1. Larger ICC values indicate higher agreement between the two delineation methods. Some scholars believe that the consistency is better when the ICC is greater than 0.75 [16]. P values of less than 0.05(*p* < 0.05) indicated statistical significance.

## Results

A total of 69 patients were enrolled in this study, 57 of whom underwent ^18^F-PSMA-1007 PET/MRI and 12 of whom underwent ^68^Ga-PSMA-11 PET/MRI. Therefore, based on different PET tracers, patients were divided into 2 groups: the ^18^F subgroup and ^68^Ga subgroup. The patients’ characteristics and volumetric results are displayed in Table 1.

**Table 1:** Patient characteristics and volumetric results

Characteristics	Median (IQR) or (%)	
	^18^F	^68^Ga
Age (years)	71(67–75)	72(68–80)
PSA (ng/ml)	15.00(7.30–30.58)	17.05(12.47–22.65)
Gleason score		
6	11(19.2)	2(16.6)
7	20(35.0)	6(50.0)
8	18(31.5)	4(33.3)
9	5(8.7)	
10	3(5.2)	
Risk classification		
Low	9(15.7)	2(16.6)
Intermediate	21(36.8)	6(50.0)
High	27(47.3)	4(33.3)
SUVmax	11.53(4.95–21.50)	10.32(5.67–18.10)
GTV-MRI, cm^3^	2.80(1.50–7.20)	2.60(1.23–4.43)
GTV-PET_VIS_, cm^3^	3.30(1.50–9.20)	1.80(0.58–2.60)
GTV-PET/MRI, cm^3^	4.80(2.12–11.55)	2.45(1.83–4.60)
GTV-PET_30%_, cm^3^	21.70(6.80–37.10)	4.10(2.23–27.85)
GTV-PET_40%_, cm^3^	10.60(3.60–19.65)	2.30(1.13–21.30)
GTV-PET_50%_, cm^3^	5.30(1.80–10.95)	2.85(0.68–14.45)
GTV-PET_60%_, cm^3^	2.40(0.70–6.25)	0.80(0.33–6.18)

*IQR* interquartile range, *GTV* gross tumor volume, *ICC* intra-class correlation coefficient

The GTV-MRI, GTV-PET_VIS_, and GTV-PET/MRI delineated by the visual method from different observers are shown in Table 2. The DSC between GTVs delineated by observers A, B, and C is displayed in Table 3. There were no significant differences in tumor volume delineated by different observers.

**Table 2:** GTVs obtained by three observers in ^18^F and ^68^Ga subgroups

Observers	^18^F			^68^Ga		
	GTV-MRI (cm^3^)	GTV-PET_VIS_ (cm^3^)	GTV-PET/MRI (cm^3^)	GTV-MRI (cm^3^)	GTV-PET_VIS_ (cm^3^)	GTV-PET/MRI (cm^3^)
A	2.70(1.30–6.20)	2.90(1.20–7.65)	4.90(1.40–10.40)	2.70(0.680–3.75)	1.60(0.43–3.55)	3.15(1.28–6.25)
B	2.90(1.40–7.90)	2.80(1.65–10.05)	4.80(2.50–11.05)	2.45(1.30–5.18)	1.30(0.70–2.78)	2.05(1.43–3.90)
C	3.40(1.25–7.15)	3.20(1.55–9.90)	5.00(2.15–12.80)	2.45(1.55–4.80)	1.70(0.75–3.20)	2.70(2.13–3.70)
	*F* = 0.176 *p* = 0.839	*F* = 0.075* p* = 0.928	*F* = 0.035 *p* = 0.965	*F* = 0.073 *p* = 0.930	*F* = 0.022 *p* = 0.978	*F* = 0.019 *p* = 0.981

*GTV* gross tumor volume

**Table 3:** DSC between GTVs delineated by observers A, B and C in the ^18^F and ^68^Ga subgroups

GTV	^18^F			^68^Ga		
	DSC(A-B)	DSC(B-C)	DSC(A-C)	DSC(A-B)	DSC(B-C)	DSC(A-C)
GTV-MRI	0.86(0.81–0.89)	0.86(0.84–0.89)	0.82(0.78-0.86)	0.90(0.86–0.95)	0.87(0.83–0.91)	0.86(0.84–0.87)
GTV-PET_VIS_	0.90(0.86–0.94)	0.87(0.85–0.90)	0.85(0.82–0.87)	0.87(0.83–0.91)	0.86(0.82–0.88)	0.82(0.78–0.86)
GTV-PET/MRI	0.91(0.87–0.94)	0.93(0.89–0.97)	0.92(0.87–0.95)	0.91(0.88–0.93)	0.96(0.92–0.97)	0.89(0.87–0.91)

*GTV* gross tumor volume, *DSC* Dice similarity coefficient, DSC(A-B), DSC between GTVs delineated by observers A and B respectively, *DSC(B-C)* DSC between GTVs delineated by observers B and C respectively, *DSC(A-C)* DSC between GTVs delineated by observers A and C respectively

### ^*18*^*F subgroup results*

**Fig. 3:**
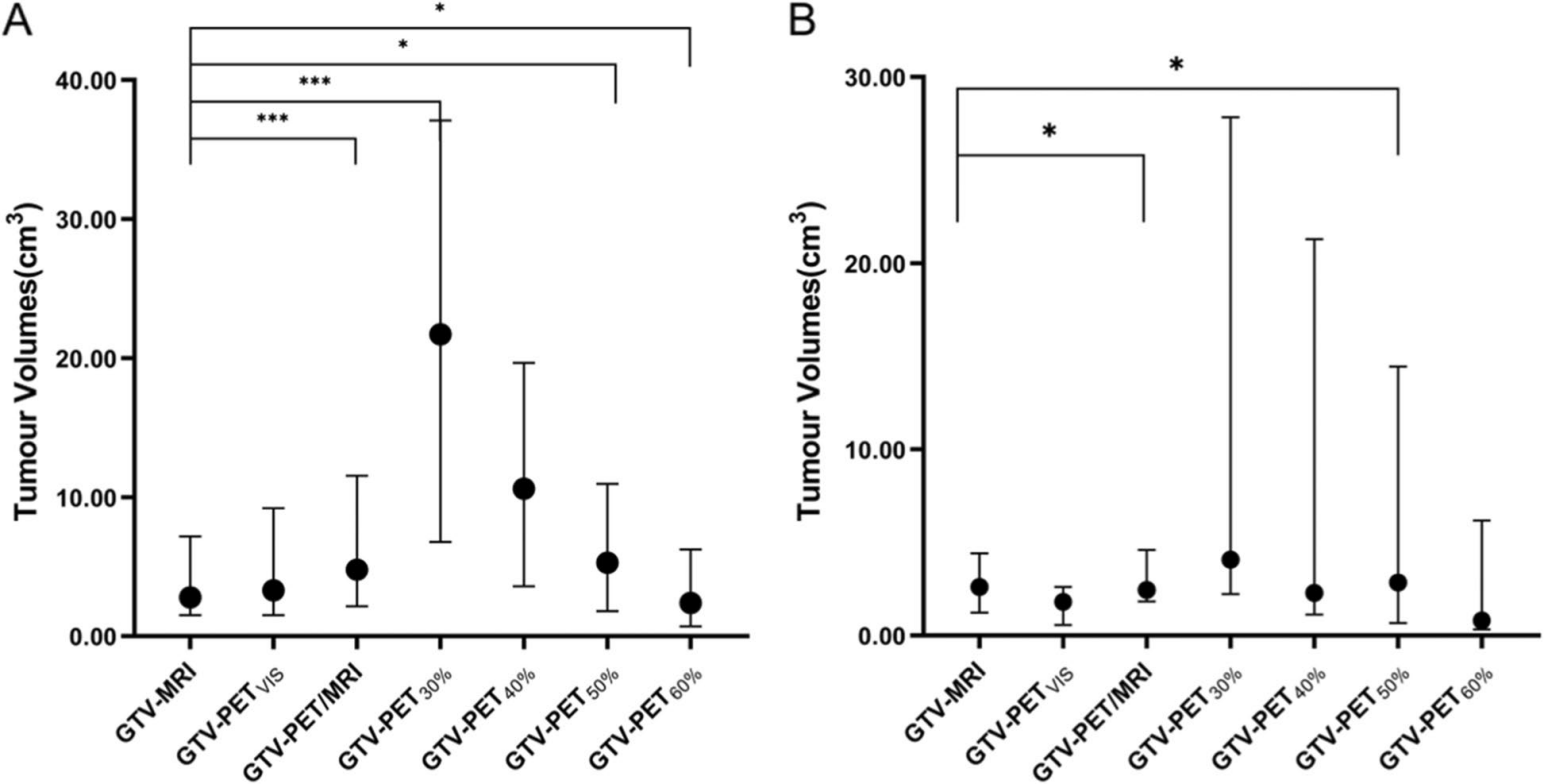
The GTV volumes of the ^18^F subgroup (**A**) and the ^68^Ga subgroup (**B**). Each item and its longitudinal extension represent the median (IQR) of the GTVs. *p < 0.05, *** *p* < 0.001

Statistical analysis showed that GTV-PET_50%_ (*r* = 0.338, *p* < 0.05), GTV-PET_60%_ (*r* = 0.317, *p* < 0.05), GTV-PET_VIS_ (*r* = 0.742, *p* < 0.001) and GTV-PET/MRI (*r* = 0.923, *p* < 0.001) were significantly related to the reference GTV-MRI. GTV-PET/MRI (*p* < 0.001), GTV-PET_30%_ (*p* < 0.001), GTV-PET_40%_ (*p* < 0.05), and GTV-PET_60%_ (*p* < 0.05) diverged such that differences were statistically significant from the referenced GTV-MRI results (Fig. 3, A).

**Fig. 4:**
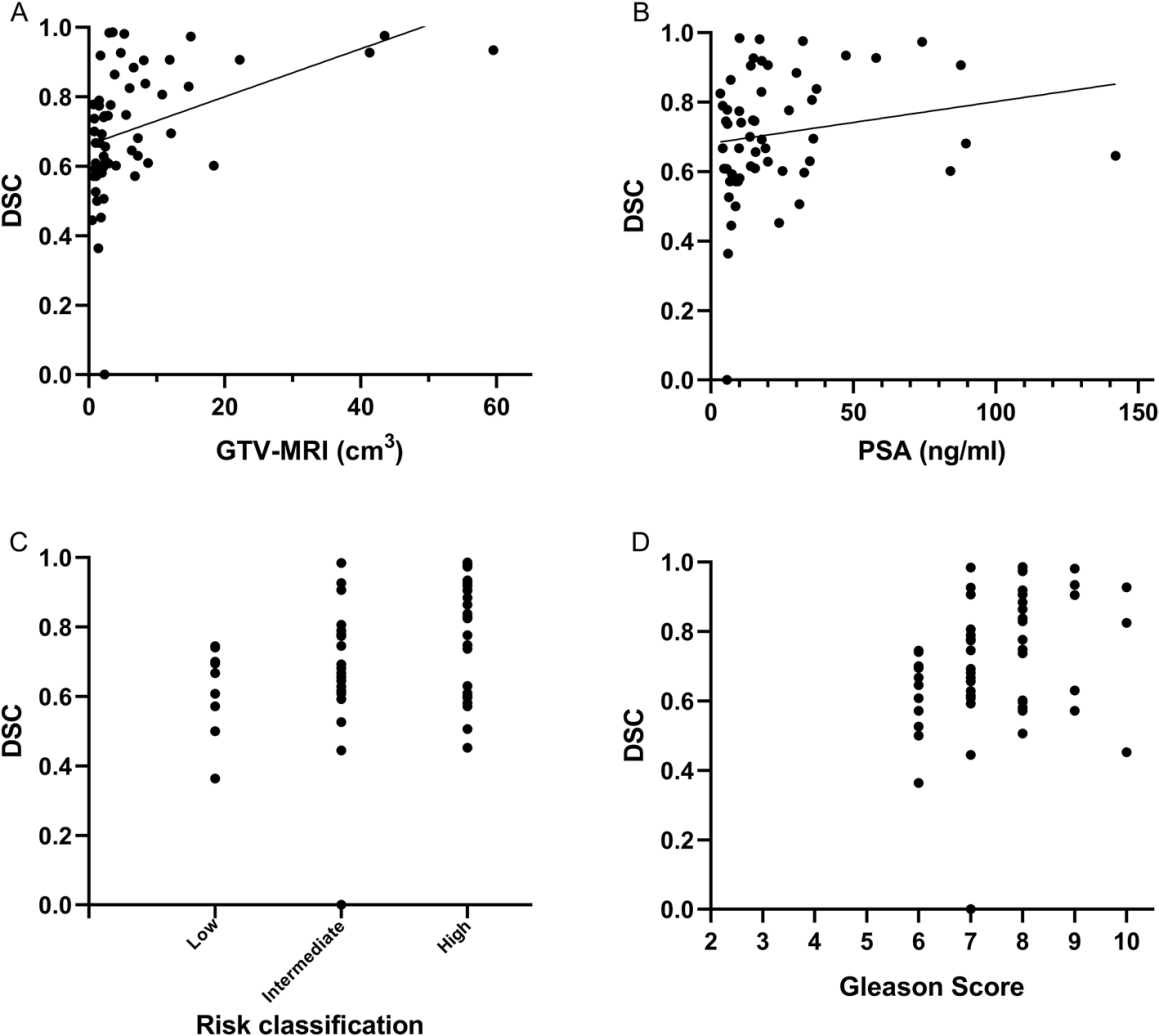
The correlation between DSC (between GTV-MRI and GTV-PET/MRI) and the reference GTV-MRI (*r* = 0.516, *p* < 0.001), PSA (*r* = 0.288, *p* < 0.05), risk classification (*r* = 0.287, *p* < 0.05), and Gleason score (*r* = 0.321, *p* < 0.05) in the ^18^F subgroup

The average DSC between GTV-MRI and GTV-PET_VIS_ was 0.45 (range 0–0.86), that between GTV-MRI and GTV-PET/MRI was 0.71 (range 0–0.99), that between GTV-MRI and GTV-PET_30%_ was 0.32 (range 0–0.80), that between GTV-MRI and GTV-PET_40%_ was 0.33 (range 0–0.75), that between GTV-MRI and GTV-PET_50%_ was 0.30 (range 0–0.71), and that between GTV-MRI and GTV-PET_60%_ was 0.23 (range 0–0.70). We selected the DSC between GTV-MRI and GTV-PET/MRI, since it was greater than 0.7, to evaluate the correlation with the reference GTV-MRI (*r* = 0.516, *p* < 0.001), PSA (*r* = 0.288, *p* < 0.05), risk classification (*r* = 0.287, *p* < 0.05), and Gleason score (*r* = 0.321, *p* < 0.05) (Fig. 4). GTV-MRI significantly and positively affected the DSC (*r*^2^ = 0.462, *p* < 0.05). Age, PSA level, Gleason score, SUVmax, and risk classification showed no linear correlation with the degree of concordance between the imaging modalities.

A total of 40 patients in the ^18^F group underwent radical prostatectomy within one month after the PET/MRI scan. The Lpath, L_PET/MRI_, L_MRI_, L_PET_, L_PET30%_, L_PET40%_, L_PET50%_ and L_PET60%_ were 1.74 (1.34–2.31) cm, 1.75 (1.41–2.41) cm, 1.41 (1.05–1.92) cm, 1.55 (1.15–2.00) cm, 2.68 (1.94–3.32) cm, 2.10 (1.49–2.59) cm, 1.64 (1.24–2.27) cm, and 1.41 (0.84–1.73) cm, respectively.

The results showed that L_PET/MRI_ (*r* = 0.893, *p* < 0.001), L_MRI_ (*r* = 0.797, *p* < 0.001), and L_PET_ (*r* = 0.888, *p* < 0.001) were significantly correlated with the gold standard Lpath. L_PET/MRI_ had the highest consistency with the gold standard Lpath (ICC = 0.893, 95% CI: 0.806–0.942, *p* < 0.001) (Table 4). According to the Bland–Altman diagram, the mean of the difference between L_PET/MRI_ and Lpath was -0.029, the 95% limit of agreement was -0.673 ~ 0.616, and only one case was outside the limit, which indicates a good consistency level of these data (Fig. 6, A).

**Table 4:** ICC consistency between Lpath and tumor lengths obtained by different methods

Length	^18^F			^68^Ga		
	ICC	95%CI	*p*	ICC	95%CI	*p*
L_PET/MRI_	0.893	0.806 ~ 0.942	< 0.001	0.837	0.505 ~ 0.953	< 0.001
L_MRI_	0.797	0.648 ~ 0.887	< 0.001	0.825	0.475 ~ 0.950	< 0.001
L_PET_	0.884	0.792 ~ 0.937	< 0.001	0.750	0.306 ~ 0.926	< 0.01
L_PET30%_	0.243	-0.071 ~ 0.513	0.063	0.467	-0.149 ~ 0.822	0.063
L_PET40%_	0.112	-0.203 ~ 0.406	0.243	0.683	0.176 ~ 0.904	< 0.01
L_PET50%_	0.211	-0.104 ~ 0.487	0.093	0.620	0.069 ~ 0.881	< 0.05
L_PET60%_	0.176	-0.140 ~ 0.459	0.135	0.575	-0.002 ~ 0.864	< 0.05

### ^*68*^*Ga subgroup results*

Statistical analysis showed that GTV-PET_30%_ (*r* = 0.629, *p* < 0.05), GTV-PET_40%_ (*r* = 0.581, *p* < 0.05), GTV-PET_VIS_ (*r* = 0.595, *p* < 0.05) and GTV-PET/MRI (*r* = 0.944, *p* < 0.001) were significantly related to the reference GTV-MRI. GTV-PET/MRI (*p* < 0.05) and GTV-PET_30%_ (*p* < 0.05) diverged statistically significantly from the reference GTV-MRI results (Fig. 3, B).

**Fig. 5:**
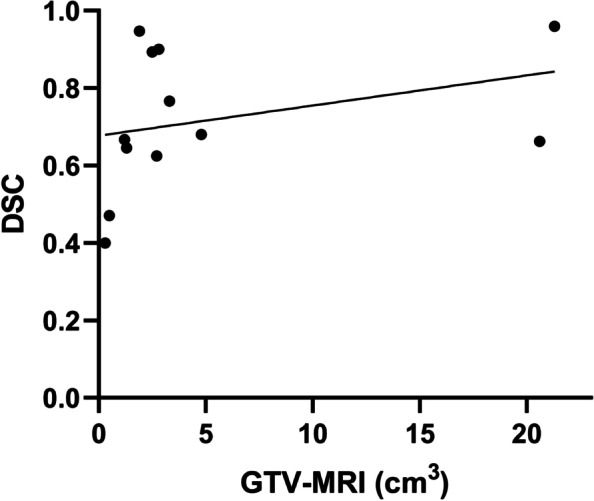
The correlation between DSC (between GTV-MRI and GTV-PET/MRI) and the reference GTV-MRI (*r* = 0.580, *p* < 0.05) in the ^68^Ga subgroup

The average DSC value between GTV-MRI and GTV-PET_VIS_ was 0.33 (range 0.08–0.55), that between GTV-MRI and GTV-PET/MRI was 0.72 (range 0.4–0.96), that between GTV-MRI and GTV-PET_30%_ was 0.33 (range 0.09–0.82), that between GTV-MRI and GTV-PET_40%_ was 0.33 (range 0.06–0.73), that between GTV-MRI and GTV-PET_50%_ was 0.27 (range 0.04–0.59), and that between GTV-MRI and GTV-PET_60%_ was 0.19 (range 0–0.38). We chose the DSC between GTV-MRI and GTV-PET/MRI, since it was greater than 0.7, to assess the correlation with the reference GTV-MRI (*r* = 0.580, *p* < 0.05) (Fig. 5). None of the variables was significantly associated linearly with the degree of concordance.

A total of 11 patients in the ^68^Ga group underwent radical prostatectomy within one month after the PET/MRI scan. The Lpath, L_PET/MRI_, L_MRI_, L_PET_, L_PET30%_, L_PET40%_, L_PET50%_ and L_PET60%_ were 1.61 (1.38–1.99) cm, 1.37 (1.24–1.29) cm, 1.33 (1.02–2.07) cm, 1.32 (1.10–2.11) cm, 1.54 (1.32–3.22) cm, 1.41 (1.11–2.69) cm, 1.54 (0.74–2.45) cm, 1.21 (0.69–1.78) cm, respectively.

**Fig. 6:**
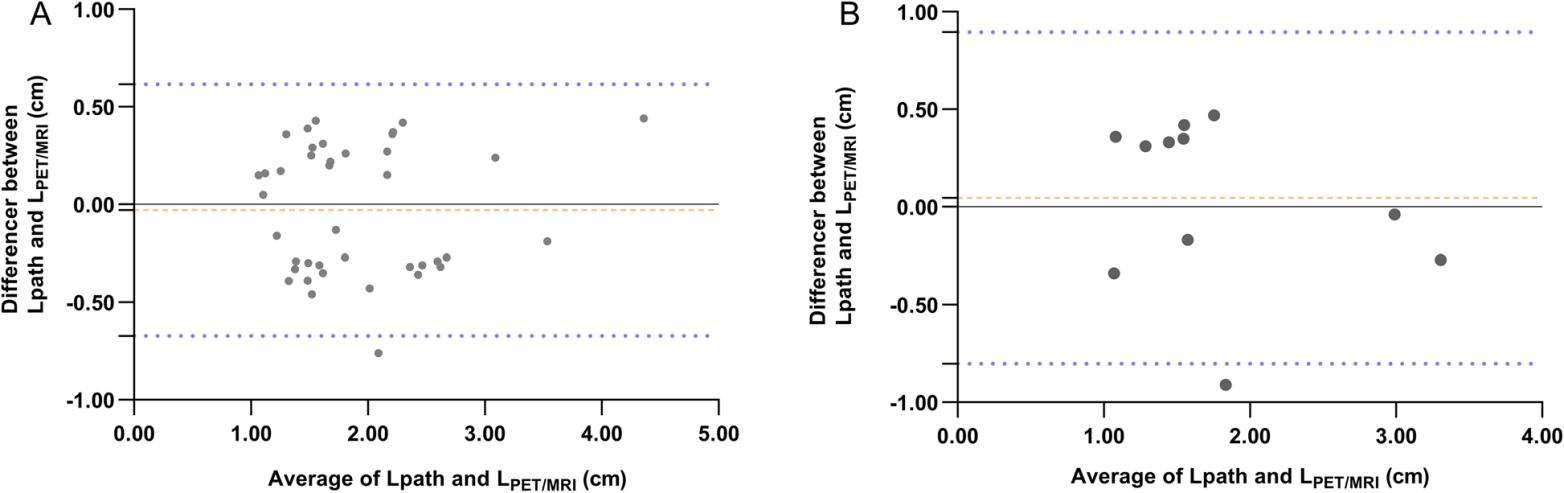
Bland–Altman plots of L_PET/MRI_ and Lpath in the ^18^F subgroup (**A**) and the ^68^Ga subgroup (**B**)

The results showed that L_PET/MRI_ (*r* = 0.848, *p* < 0.001), L_MRI_ (*r* = 0.831, *p* < 0.01), L_PET_ (*r* = 0.761, *p* < 0.01), L_PET40%_ (*r* = 0.717, *p* < 0.05) and L_PET50%_ (*r* = 0.639, *p* < 0.05) were significantly correlated with the gold standard Lpath. L_PET/MRI_ had the highest consistency with the gold standard Lpath (ICC = 0.837, 95% CI: 0.505–0.953, *p* < 0.001) (Table 4). According to the Bland–Altman diagram, the mean of the difference between L_PET/MRI_ and Lpath was 0.046, the 95% limit of agreement was -0.803 ~ 0.896, and only one case was outside the limit, which indicates a good consistency level of these data (Fig. 6, B).

## Discussion

PCa is a radiation-sensitive tumor and the development of small-molecule PSMA inhibitors has rendered PSMA ligand-coupled radionuclide diagnosis and treatment of PCa a research hotspot. Currently, the most commonly used method for GTV delineation is the visual method, which has low technical requirements but high observer dependence. To examine the reproducibility of the visual method, we specifically assessed the interobserver variability. The results showed no significant differences between observers in either the ^18^F subgroup or ^68^Ga subgroup, especially based on hybrid PET/MRI images. On this basis, we took the mean of delineated GTV volumes from three observers for further study.

We compared the differences in the delineation of GTVs based on MRI, PET, and hybrid PET/MRI. GTV-PET/MRI was significantly related to the reference GTV-MRI, and there were also statistically significant differences in both the ^18^F and ^68^Ga subgroups. Our results also showed that all DSC values between GTV-MRI and GTV-PET/MRI were above 0.7, indicating high spatial overlap. We initially concluded that GTV delineation based on PET/MRI is better than that based on PET images alone. We then validated this hypothesis with pathology. L_PET/MRI_ was most correlated with Lpath in both the ^18^F and ^68^Ga subgroup. Moreover, both ICC consistency and Bland–Altman analysis showed that GTV delineation based on fusion PET/MRI was superior to other methods. Considering the above results, we believe that GTV delineation based on fusion PET/MRI is more accurate. A possible reason is that simultaneous PET/MRI synthesizes metabolic and morphological features to reveal the characteristics of tumor lesions and distinguish the border from surrounding normal tissues better than either method alone. Some scholars also concluded that delineating GTV on hybrid PET/MRI is feasible and that the combination of PET/MRI provides more information during GTV delineation in radiotherapy planning than other standard imaging methods, which is consistent with our results despite the different tumor locations [17, 18]. The conventional standard for PCa is mpMRI; however, Johnsen et al. concluded that mpMRI is limited in its ability to detect PCa foci, missing approximately one-third of clinically significant foci [19]. One study demonstrated the usefulness of PSMA-based PET and suggested that it could be used in combination with MRI to improve clinically significant PCa detection [20]. We also recommend GTV delineation on fused PET/MRI images.

Our results showed that 91.2% of GTV-PET/MRI outcomes were greater than those of GTV-MRI in the ^18^F subgroup and 83.3% of GTV-PET/MRI outcomes were greater than those of GTV-MRI in the ^68^Ga subgroup. In a previous study [21], it was also argued that MRI-based profiles generally underestimate tumor size. Experts, therefore, recommend that adequate radiation doses should be maintained for the entire prostate in focal radiotherapy to prevent undertreatment in areas with no tumor detected tumor. Gibson et al. recommended that an enlarged GTV margin defined by mpMRI may support focus-enhancing or therapeutic PCa [22]. Several studies of different tumor sites have also shown that that GTV based on PET/MRI is larger than that obtained from GTV-MRI because of the additional biological information provided by PET, which can reduce the risk of locational errors and affect GTV variation during radiotherapy, thus influencing the radiation dose administered to normal tissue [17, 23, 24]. Therefore, we hypothesized that a larger GTV-PET/MRI may result in the acquisition of a more complete target, allowing adequate irradiation of the lesion, and, thus, affecting the therapeutic effect.

For the results obtained by the threshold method for delineating target areas, the 60% SUVmax for ^18^F-PSMA-PET and 30% SUVmax for ^68^Ga-PSMA-PET seemed to be positive results, but the spatial overlap between the GTVs delineated with these thresholds and the reference GTV-MRI was very low. Therefore, the volume difference was significant, and the optimal threshold cannot be comprehensively determined. This may be attributed to the low spatial resolution of PET. Due to the limitation of the positron range in the process of developing PET detectors, it is difficult to improve the resolution of PET images, which is likely to lead to the blurring of tumor edges on PET images. The partial volume effect can reduce the SUV value of PET images to some extent, which will lead to blurred tumor edges and result in errors in diagnosis and target delineation. To date, there are few studies on the threshold method and no unified threshold standard has been formed. However, referable conclusions have been reached in some other studies; for example, Alfano et al. showed that the sensitivity could be improved to 95.0 ± 7.8% with a threshold of 67% SUVmax. Furthermore, the specificity could be improved to 95.1 ± 5.2% with a threshold of 81% SUVmax by histopathological verification [25]. Another study, using the histopathological GTV (GTVhisto) as the gold standard reference, revealed that the median statistically optimized SUV% threshold of 41 SUV% for ^68^Ga-PSMA-11 PET and 44 SUV% for ^18^F-PSMA-1007 PET form a basis for tracer-specific window leveling [26]. Different tracers lead to different SUV% distributions throughout the prostate, which also suggests that the threshold should not be generalized. Therefore, considering the limitations of PET, we emphasize the GTV delineation on the PET/MRI fusion images. As Zamboglou et al. [8]. demonstrated, using a fusion image (mpMRI ∪ PSMA PET) to delineate GTV achieves the highest sensitivity and spatial overlap compared with PSMA PET or MRI-based GTV delineations alone.

Since the DSC between GTV-MRI and GTV-PET/MRI was greater than 0.70, we investigated the correlation with the reference GTV-MRI, age, SUVmax, risk classification, Gleason score, and PSA. All of the above variables were significantly correlated with DSC in the ^18^F subgroup, and GTV-MRI was the influencing factor for DSC. In the ^68^Ga subgroup, only GTV-MRI was significantly correlated with DSC. As a previous study showed, the spatial overlap between GTV-MRI and GTV-PET/MRI increases as the tumor volume increases [23]. Draulans C et al. investigated the effect of tracer variability on the GTV profile and found that the distribution of SUV% in the prostate was significantly altered in ^18^F-PSMA-based images, resulting in increased interobserver variability [27]. It has already been confirmed that PSMA is associated with a higher Gleason score, and PSMA expression increased with Gleason score [28]. As previously reported [29], increased PSA levels before scanning were associated with increased PSMA positivity. Clinically, comprehensive T staging, Gleason score, and PSA level were used to evaluate the risk classification of PCa. The combination of these parameters is an important indicator in an evaluation of the degree of malignancy [30].

Furthermore, the different results of the two groups were not only related to the relatively low number of patients in the ^68^Ga group but also possibly related to different tracer characteristics. High tracer retention in the bladder and ureter is known to lead to errors in image analysis [31]. Compared with ^68^Ga-PSMA-11, ^18^F-PSMA-1007 excretion passes mainly through hepatobiliary channels rather than the urine, giving it a superior advantage in focal detection given the lower uptake of imaging agents in periprostatic normal tissue and more pronounced contrast to normal tissue [27, 32]. In the future, a direct comparison of the accuracy of ^18^F-PSMA-1007 and ^68^Ga-PSMA-11 PET/MRI in GTV delineation needs to be conducted.

A major limitation of this study was the relatively small number of patients, especially in the ^68^Ga group. Due to the short development time of PSMA PET, especially in the clinical application of ^68^Ga-PSMA PET/MRI and ^18^F-PSMA PET/MRI, studies with large samples are still lacking. Furthermore, although the prognosis of radiotherapy based on PET/MRI GTV delineation is uncertain, many studies have shown that it has an impact on target delineation. Accordingly, we can speculate that it has different clinical effects on PCa patients treated with radiotherapy. There is still an urgent need for more prospective, multicentre, large-sample, prospective studies.

## Conclusion

It is feasible to visually delineate GTV on PSMA PET/MRI in PCa radiotherapy, and we emphasize the GTV delineation on the PET/MRI fusion image. In addition, the overlap degree was the highest between GTV-MRI and GTV-PET/MRI, and it increased with increasing tumor volume. These results may have important implications for the assessment and treatment of PCa. PSMA PET/MRI is promising in the development of more accurate and personalized tumor radiotherapy.

## Data Availability

The data used to support the findings of this study are available from the corresponding author upon request.
